# Calibration of a Monitor for Use in Bremsstrahlung Beams

**DOI:** 10.6028/jres.065A.040

**Published:** 1961-10-01

**Authors:** E. G. Fuller, Evans Hayward

## Abstract

The calibration of a thick-walled ionization chamber by means of a sodium iodide scintillation spectrometer is described. The calibration was made for six bremsstrahlung energies in the range 6 to 19 Mev.

## 1. Introduction

A thick-walled, parallel-plate, aluminum ionization chamber has been designed and constructed [1]^1^ as a standard to measure the X-ray intensity of the bremsstrahlung beams of betatrons and synchrotrons. This chamber is of simple, rugged construction and presumably can be reproduced in any laboratory. It has already been calibrated for peak bremsstrahlung energies in the range 20 to 180 Mev by caliormetry [1] and by means of a scintillation spectrometer [2]. The present paper describes its calibration by the latter method [3] for bremsstrahlung energies in the range 6 to 19 Mev. This calibration then permits for the first time the measurement in different laboratories of photonuclear cross sections in the giant resonance regions with reference to the same monitor.

## 2. Method

**Figure 1:**
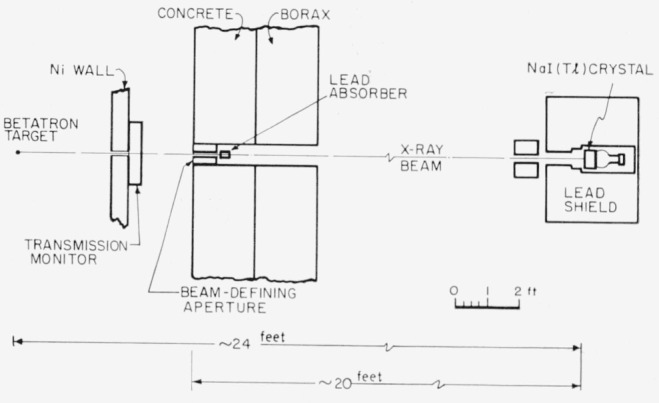
Geometry of the experiment.

The object of this calibration was to relate the number of coulombs collected from the thick-walled ionization chamber to the amount of energy in the bremsstrahlung beam incident on it. This calibration was performed in two steps. First, the reading on a transmission monitor (see fig. 1) was related to the charge collected from the standard chamber by taking the ratios of their readings. For this comparison the standard chamber was carefully located in the betatron bremsstrahlung beam in the position later occupied by a sodium iodide scintillation spectrometer. The second step was to use the spectrometer to relate the amount of energy in this beam to the reading of the transmission monitor. These two measurements then yield the number of coulombs collected from the standard chamber per Mev of bremsstrahlung energy incident on it for the beam filtration equivalent to 7.25 g/cm^2^ of aluminum normally used with this betatron. In a subsidiary experiment the effects of filtration were studied by varying the aluminum thickness between a thin transmission chamber and the standard chamber.

The relationship between the amount of energy incident on the scintillation spectrometer and the charge collected from the transmission ionization chamber was determined by measuring the total number of interactions in the crystal produced by the beam. For this measurement a thick absorber was placed between the transmission ionization chamber and the crystal. (See fig. 1.) This absorber was introduced to reduce the intensity of the X-ray beam at the crystal so that it could be counted without pulse pile-up and at the same time accurately monitored by the ionization chamber. The absorber also removed the lowest energy photons from the beam, making the pulse height distributions obtained more amenable to interpretation. In the previous calibration [2] that employed this method carbon absorbers were used. This approach was not possible in the work reported here, because the physical dimensions of the experimental area in front of the betatron are not great enough to provide the good geometry required. It has already been shown [4], however, that it is possible to calibrate this beam using lead absorbers for which the Compton scattering cross section is much less important and geometrical considerations are therefore minimized. Lead absorbers were therefore used in the final calibration.

The physical arrangement is shown in figure 1. The bremsstrahlung beam traversed the betatron donut wall and the parallel-plate transmission chamber before being defined by a small hole ( 116 in. in diameter) in an 8-in. long lead block. This aperture produced a beam approximately 2 in. in diameter at the back of the experimental area about 24 ft from the betatron target. Before reaching the sodium iodide crystal this beam passed through but did not strike a secondary aperture that removed stray radiation. The absorbers were placed in the beam just after the beam-defining aperture.

The use of a scintillation spectrometer to calibrate a bremsstrahlung beam depends on the ability to relate the total number of photons materialized in the sodium iodide crystal to the amount of energy in the beam. This calibration is based on a knowledge of the X-ray attenuation coefficients for all the absorbing materials in the beam, as well as for the sodium iodide itself. The assumed shape of the bremsstrahlung spectrum, *I*(*E*_0_,*E*), is of less importance. If *A*(*E*_0_) = *∫ dE I*(*E*_0_,*E*) represents a unit of bremsstrahlung energy leaving the betatron target lying in the cone transmitted by the beam-defining aperture when electrons of energy *E*_0_ strike the internal betatron target, then the number of photons materialized in the sodium iodide crystal is

$$P(L,E_{0})=\int dE\frac{I(E_{0},E)}{E}e^{-L(E)}[1-e^{-T(E)}].$$ (1)

The symbol *L*(*E*) represents the number of mean free paths of material between the bremsstrahlung-producing target and the detector and *T*(*E*) the number in the sodium iodide crystal itself. At the same time, the amount of energy passing through the beam-defining aperture is

$$A^{\prime }(E_{0})=\int dEI(E_{0},E)e^{-L^{\prime }(E)},$$ (2)

where *L*′ (*E*) is the number of mean free paths of absorber in the donut wall and the transmission monitor. If *C*(*L,E*_0_) stands for the total number of spectrometer counts registered per volt measured on the transmission monitor, and *R*(*E*_0_) is the number of coulombs collected from the standard chamber per volt measured on the same monitor, then the calibration of the standard chamber in coulombs/Mev is

$$\alpha (E_{0})=\frac{R(E_{0})}{C(L,E_{0})}\frac{P(L,E_{0})}{A^{\prime }(E_{0})}.$$ (3)

This method is based on the assumption that each photon interacting in the absorbing material results in its removal from the beam. The applicability of this assumption can be checked experimentally by performing the calibration for several values of absorber thickness, *L*. This test was used as the criterion of acceptability of the results. A valid calibration must be independent of *L*.

## 3. Details of the Measurement

The scintillation spectrometer consisted of a NaI(Tl) crystal and associated electronics. The sodium iodide crystal was 5 in. in diameter and 4 in. long and was viewed by a 5-in. photo-multiplier tube. This assembly was encased in the lead box shown in figure 1. The pulses were amplified and analyzed by a 20-channel pulse height analyzer. The analyzer was gated so as to accept pulses only during a 25-*µ*sec interval around the betatron yield pulse. To avoid pulse pile-up the counting rates were at all times maintained at less than five counts per second for all pulses above a bias corresponding to the absorption of ~300 kev by the crystal. The repetition rate of the betatron was 180 pulses/sec.

The charge collected from the transmission monitor (and from the standard chamber as well) was determined by measuring the voltage developed across a polystyrene film capacitor. This voltage was cancelled by that supplied and measured by a potentiometer. The null condition was established with a vibrating reed electrometer. The capacitors were calibrated against a standard, using a bridge operated at 1,000 cycles. The resulting errors in the absolute charge measurements are small compared to those inherent in the determination of the absolute number of counts in the sodium iodide crystal.

The data to be reported here were taken in a two-week period during which the following measurements were made as a daily routine: (1) The sensitivity of the transmission chamber, which was closed to the atmosphere, was checked by measuring the time required to charge a capacitor to a standard voltage when a radioactive source was placed in a standard position. This reading was reproducible to within 0.5 percent. (2) The leak-rate of this monitor was measured. It was large enough to produce corrections to the monitor reading varying from 0.1 to 2 percent. (3) The stability of the counting equipment was checked by measuring the pulse height distribution produced by a Na^22^ source and by using a pulser to determine the bias of the top and bottom of the pulse height analyzer. The overall gain of the system changed by less than 2 percent. This variation has a negligible effect on the calibration.

The absorbers used in this calibration were lead disks 2 in. in diameter and 1 in. long. They were carefully machined so that their thickness in g/cm^2^ could be determined by weighing and accurately measuring their diameters. They were placed in the beam just after the beam-defining aperture. The choice of absorber thickness was determined empirically. It was found that at least 5 in. of lead were required between the transmission monitor and the spectrometer to reduce the X-ray intensity at the crystal enough so that the counting could be done without pulse pile-up, and to maintain a high enough intensity at the ionization chamber to make a reliable measurement possible. The upper limit on the thickness was set at 7 in. by the magnitude of the multiple scattering in the absorber. The use of eq (1) to calculate the number of photons materialized in the sodium iodide crystal assumes that every interaction of a photon in the absorber results in its removal from the beam. As will be seen in the following, for sufficiently thick absorbers the buildup of secondaries is great enough to make this assumption invalid.

At each of the six bremsstrahlung energies used in this calibration, the pulse height distributions produced by the photons transmitted by 5, 6, 7, and 8 in. of lead were recorded. Two runs were made under each condition to yield a total of 10^4^ counts. The statistical uncertainty of the calibration is therefore of the order of 1 percent.

The principal source of background was that resulting from cosmic rays and general background radioactivity. This was determined by measuring the counting rate with the X-rays off and the pulse height analyzer ungated. The background for each run was then determined by multiplying this counting rate by the time for each run and reducing by the duty cycle of 4.6×10^−3^. An attempt was made to evaluate the betatron-produced backgrounds. For this purpose the number of counts registered per monitor reading was recorded when an additional 8-in. lead barrier was placed in front of the sodium iodide crystal. These measurements were made at selected bremsstrahlung energies and for various absorber thicknesses, *L*(*E*), in the incident beam. These yields were found to be negligible compared with those measured with the 8 in. removed, indicating that there was no serious contribution to the background from this source.

## 4. Treatment of the Data

**Figure 2:**
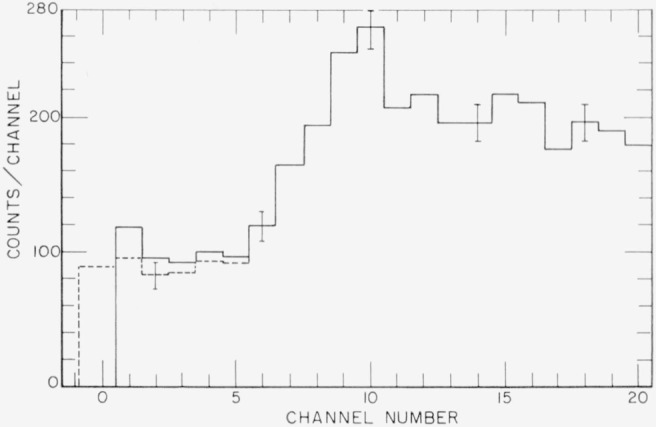
Pulse height distribution produced when the 13-Mev bremsstrahlung spectrum traversed 6 in. of lead. In this spectrum there were an additional 1815 counts registered above the top of the analyzer. The errors indicated are based on the number of counts. The dotted histogram in the first five channels has been corrected for the cosmic ray background. The dotted area below the first channel is an extrapolation and was taken to represent the number of pulses too small to be recorded in the analyzer.

A typical pulse height distribution is shown in figure 2. It was obtained using a 13 Mev bremsstrahlung spectrum and 6 in. of lead absorber in the beam. As a result of the steep energy dependence of the lead X-ray attenuation coefficient, the spectrum of transmitted photons consists of a broad maximum peaking near 3 Mev, i.e., channel 10. An appreciable fraction (1,815 counts in this case) of the distribution lies above the top of the analyzer. The flat part of the distribution below channel 6 results from the incomplete absorption of higher energy photons in the sodium iodide crystal. The dotted histogram in the first five channels has been corrected for background. The dotted area that extends 1.38 channels below the threshold of the pulse height analyzer is an extrapolation of the distribution to include the pulses too small to be recorded. The total number of counts in the distribution then was obtained by taking the number above the threshold of the analyzer, subtracting the gated room background and adding a correction for the pulses below the threshold. The two corrections are small and tend to cancel so that the total correction was usually less than 1 percent.

The results are presented in table 1. The quantities, *C*(*L,E*_0_) and *R*(*E*_0_), are the experimental data. The ratios, *P*(*L,E*_0_)/*A*′(*E*_0_), were obtained by evaluating the integrals of eqs (1) and (2). The bremmstrahlung spectra were obtained from the tabulation of Penfold and Leiss [5] and the X-ray attenuation coefficients from the tabulation of White [6]. The absorption by the donut wall, equivalent to 1.59 g/cm^2^ of aluminum, the transmission monitor 5.66 g/cm^2^ of aluminum, and the thick lead absorbers were all taken into account. The values in parentheses are the result of carrying out the same integrations with the pair production cross section for lead increased by 2.5 percent. Wyckoff and Koch [7] have pointed out that this modification would be more consistent with the results of their attenuation measurements. This adjustment decreases the magnitudes of the integrals by only 0.5 percent. Reasonable changes in the shape of the assumed bremsstrahlung spectrum have an even smaller effect.

The calibrations of the standard chamber, *α* (*E*_0_), obtained using the four different lead absorber thicknesses are also given in the table. At each energy the value of *α* (*E*_0_) obtained using the 8-in. lead absorber is the smallest, and it differs from the average by the largest amount. This systematic trend was taken as an indication that a small fraction of the photons detected by the spectrometer had already been scattered and that the assumptions underlying the calibration procedure were failing. Only the calibrations obtained using 5, 6, and 7 in. of lead as absorbers have therefore been included in the final average. The errors quoted in the table are based on the internal consistency of these three numbers.

The effect of secondary processes on the calibration was even more dramatic when carbon absorbers were used. The calibrations were inconsistent for any pair of thicknesses differing by a mean free path and great enough to make the ion chamber measurements compatible with the scintillation counting.

The final numbers in the table represent the calibration of the standard chamber for a beam diameter of 2.25 in. and a filtration of the incident bremsstrahlung beam equivalent to 7.25 g/cm^2^ of aluminum. The calibration does not depend critically on either of these quantities. It has been shown [1] that this calibration varies with beam diameter by less than 1 percent for beam diameters up to 6 in.

In the transmission ionization chamber used for this calibration the bremsstrahlung beam passed through 5.66 g/cm^2^ of aluminum. To check the effect of filtration on the calibration this chamber was replaced by one that contained only 1.37 g/cm^2^ of aluminum, At each bremsstrahlung energy the reading of the standard chamber was compared to that of the thin transmission monitor as a function of aluminum thickness placed between them. The thicknesses used bracketed the filtration used in in the present calibration. It was found that the charge collected from the standard chamber was essentially proportional to the amount of bremsstrahlung energy incident on it, i.e., proportional to *A*′(*L′,E*_0_) calculated from eq (2), for *L*′ ranging from 2.96 (donut plus thin-walled ionization chamber) to 10 g/cm^2^ of aluminum. Deviations from proportionality as large as 2 percent may exist for the extreme thickness at the two lowest energies, 6 and 8 Mev. For reasonable thickness, then, the calibration presented here is independent of the distortions produced in the spectrum by absorbers and gives a measure of the energy incident on the monitor chamber.

**Figure 3:**
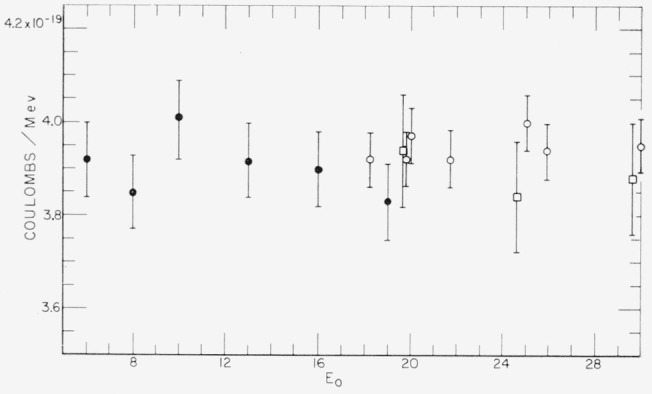
Calibration of the standard chamber in coulombs/Mev as a function of the incident bremsstrahlung energy E_0_. The closed circles are the data obtained in the calibration reported here. The open circles represent the data obtained by calorimetry and reported in [2]. The squares represent the results of Leiss, Pruitt, and Schrack [3], who made a calibration very similar to the one described here. Their data have been decreased slightly to take into account a modification of the standard chamber made since their calibration.

The results of this calibration are plotted in figure 3 as a function of bremsstrahlung energy, *E*_0_. The uncertainty in this calibration results from the statistical uncertainties (±1%), the possible errors in the methods used to subtract background and extrapolate the pulse height distribution to zero (±0.3%), the uncertainty in the determination of *R*(*E*_0_) (±1%), and the uncertainties in the evaluation of *P*(*L,E*_0_) and *A*′(*E*_0_) (±0.7%). The uncertainty of ±2 percent given in figure 3 has been made larger than the value obtained by combining these uncertainties to take into account possible unknown systematic errors. Also shown in figure 3 are the results of the other two calibrations of the standard chamber [1,2] in the energy range up to 30 Mev. It is satisfying to note that in the small energy range in which these calibrations overlap that there is good agreement in the absolute magnitude. The results of the present calibration are essentially independent of bremsstrahlung energy, *E*_0_. Only the point at *E*_0_=10 Mev fails to include the average value for the six energies of 3.905 10^−19^ coulombs/Mev within its error bar. In using this calibration it is therefore sufficient to use this single number at all energies.

## Figures and Tables

**Table 1:** 

*E*_0_	Lead thickness	*C*(*L,E*_0_)	*R*(*E*_0_)	*P*(*L, E*_0_)/*A*′(*E*_0_)	*α*(*E*_0_)	RMS deviation
						
*Mev*	*in*.	*counts/volt* ×10^3^	*coulombs/volt* ×10^−11^	*counts/Mev* ×10^−4^	*coulombs/Mev* ×10^−19^	%
6	5	129.3	9.121	5.597	$\begin{array}{l} 3.948\\ 3.983\\ 3.838 \end{array}\}3.92$	1.6
	6	40.26		1.756		
	7	12.54		0.5277		
	8	3.946		0.1615	3.733	
8	5	134.9	10.07	5.205	$\begin{array}{l} 3.891\\ 3.921\\ 3.741 \end{array}\}3.85$	2.0
	6	42.04		1.637		
	7	13.25		0.4923		
	8	4.150		0.1507	3.657	
10	5	128.6	10.83	4.775 (4.771)	$\begin{array}{l} 4.021\\ 3.962\\ 4.044 \end{array}\}4.01$	.86
	6	41.11		1.504 (1.503)		
	7	12.13		0.4529 (.4529)		
	8	3.887		0.1386 (.1388)	3.862	
13	5	119.7	11.78	3.961 (3.946)	$\begin{array}{l} 3.898\\ 3.926\\ 3.940 \end{array}\}3.92$	.45
	6	37.24		1.241 (1.236)		
	7	11.11		0.3716 (0.3704)		
	8	3.452		0.1132 (0.1130)	3.863	
16	5	107.6	12.66	3.340 (3.318)	$\begin{array}{l} 3.930\\ 3.842\\ 3.932 \end{array}\}3.90$	1.1
	6	34.30		1.041 (1.035)		
	7	10.00		0.3106 (0.3089)		
	8	3.115		0.09430 (0.09392)	3.833	
19	5	99.16	13.43	2.868 (2.845)	$\begin{array}{l} 3.884\\ 3.798\\ 3.803 \end{array}\}3.83$	1.0
	6	31.52		0.8914 (0.8847)		
	7	9.361		0.2651 (0.2633)		
	8	2.903		0.08031 (0.07990)	3.715	

## Footnotes

1 Figures in brackets indicate the literature references at the end of this paper.
